# From the ground up: biotic and abiotic features that set the course from genes to ecosystems

**DOI:** 10.1002/ece3.2468

**Published:** 2016-09-09

**Authors:** Craig W. Benkman, Sierra Jech, Matthew V. Talluto

**Affiliations:** ^1^ Department of Zoology and Physiology University of Wyoming Laramie WY USA; ^2^ Department of Botany and Chemistry University of Wyoming Laramie WY USA; ^3^ Laboratoire d'Ecologie Alpine (LECA) CNRS Université Grenoble Alpes Grenoble France

**Keywords:** foundation species, *Pinus contorta*, serotiny, *Tamiasciurus hudsonicus*, Yellowstone

## Abstract

Spatial variation in cone serotiny in Rocky Mountain lodgepole pine (*Pinus contorta* ssp. *latifolia*) across Yellowstone National Park influences initial pine recruitment after stand‐replacing fire with tremendous population, community, and ecosystem consequences. A previous study showed that much of the spatial variation in serotiny results from the balance of selection arising from high frequencies of fire favoring serotiny countered by opposing selection exerted by American red squirrels (*Tamiasciurus hudsonicus*) as seed predators. This earlier study, however, assumed stable local red squirrel densities over multiple generations of pines. Here, we examine environmental properties that might contribute to long‐term stability in the densities of red squirrels among sites. We found that the amount of clay in the soil, an indicator of plant and fungal growth—the latter an important food resource for red squirrels—and the coefficient of variation (CV) in diameter at breast height (DBH) of forest trees together account for a substantial amount of variation in red squirrel density. Soil development occurs over very long time scales, and thus, intersite variation in the amount of clay is unlikely to shift across pine generations. However, CV of DBH and squirrel density increase with stand age, which acts to amplify selection against serotiny with increasing interfire interval. Regardless, much of the variation in the CV of DBH is accounted for by soil bulk density, mean annual temperature, and surface curvature, which are unlikely to vary in their relative differences among sites over time. Consequently, these soil and abiotic attributes could contribute to consistent spatial patterns of red squirrel densities from one pine generation to the next, resulting in consistent local and spatial variation in selection exerted by red squirrels against serotiny.

## 1 Introduction

**Figure 1:**
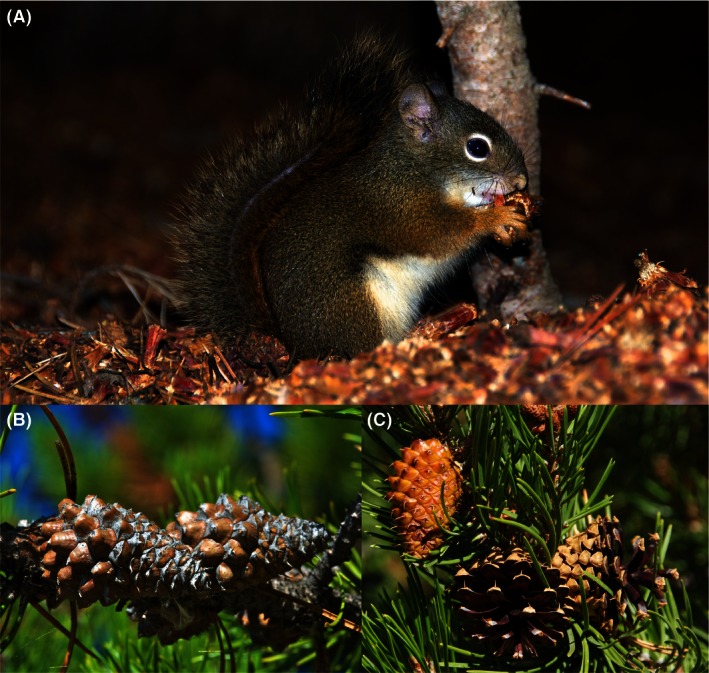
(A) A red squirrel (*Tamiasciurus hudsonicus*) eating a seed from a lodgepole pine (*Pinus contorta* ssp. *latifolia*) cone; only a few scales remain on the cone (on the distal end, directed downward). (B) Serotinous cones, which can remain closed for several decades unless removed by red squirrels or opened from heat of a fire. (C) Nonserotinous cones open in early autumn several weeks after the seeds mature; leftmost cone opened within weeks after photograph was taken. Photographs taken by C. Benkman

When we think of species having large and disproportionate impacts on communities (keystone species), apex predators that drive trophic cascades come to mind (Estes et al., 2011). For example, by eating and depleting sea urchins, sea otters prevent urchins from eating and depleting kelp (Estes et al., 2011). The huge difference between having kelp forests and their diverse community of fishes, sea lions, and eagles, versus largely kelp‐less barrens arises from contemporary ecological processes, otters directly eating urchins and indirectly facilitating the increase in kelp. Such cascading effects are thought to be widespread both on land and in water where you have strongly interacting species (Estes et al., 2011). Although less appreciated, strongly interacting predators and herbivores potentially have strong evolutionary effects on their prey (Benkman, 2013; Benkman, Siepielski, & Parchman, 2008; Steinberg, Estes, & Winter, 1995). When such prey dominates a landscape (e.g., foundation species), the ecological consequences of these evolutionary effects could be profound. Our research indicates that Rocky Mountain lodgepole pine (*Pinus contorta* ssp. *latifolia*), which dominates ~26 million ha (Lotan & Critchfield, 1990), is one such foundation species (Talluto & Benkman, 2013, 2014). The evolutionary effect is the result of differential seed predation by American red squirrels (Fig. 1A; *Tamiasciurus hudsonicus*; Talluto & Benkman, 2014).

**Figure 2:**
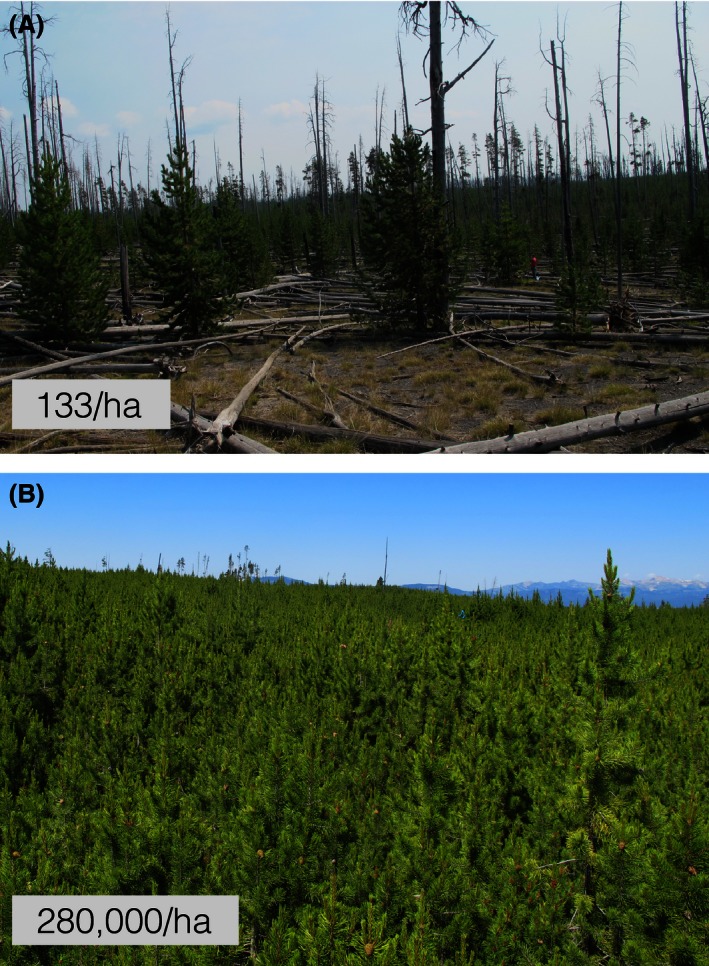
Two photographs showing range in variation of seedling densities 24 years after the 1988 fires in Yellowstone National Park. Photographs taken by K. Nelson

Earlier work on forest recovery following the 1988 Yellowstone National Park fires found that one of the main determinants of lodgepole pine seedling density in young stands was the degree of prefire cone serotiny of lodgepole pine (Tinker, Romme, Hargrove, Gardner, & Turner, 1994; Turner, Romme, Reed, & Tuskan, 2003). Serotiny occurs when woody plants encase their seeds for multiple years in woody structures (e.g., hard woody cones in lodgepole pine; Fig. 1B) creating an arboreal seed bank, which is released soon after a stand‐replacing fire (Lamont, Le Maitre, Cowling, & Enright, 1991). When the frequency of serotiny among the pines is high, large numbers of seeds are released resulting in large numbers of seedlings. Lower frequencies of serotiny result in fewer seeds available after a fire and many fewer seedlings. Because variation in seedling density among locations in Yellowstone was so great (range: 0–535,000 seedlings/ha; Turner, Tinker, Romme, Kashian, & Litton, 2004; Fig. 2), there were tremendous consequences of this variation for various ecosystem processes and plant populations (Kashian, Turner, & Romme, 2005; Turner, Romme, & Tinker, 2003; Turner, Whitby, Tinker, & Romme, 2016; Turner et al., 2004) and, undoubtedly, for numerous animal populations including birds (R. Hutto, pers. comm.). Accordingly, understanding the causes of spatial variation in serotiny has been a long‐standing goal.

Most previous research on landscape‐level variation in serotiny has focused on the importance of variation in fire frequency (Gauthier, Bergeron, & Simon, 1996; Lamont & Enright, 2000; Radeloff, Mladenoff, Guries, & Boyce, 2004). In fact, the differences in serotiny for lodgepole pine between lower and higher elevations of Yellowstone are consistent with variation in fire frequency (Schoennagel, Turner, & Romme, 2003; Talluto & Benkman, 2013, 2014). Nevertheless, theory and observation suggest that seed predation, especially where seed predators kill a large fraction of the seeds, can select against serotiny (Benkman & Siepielski, 2004; Enright, Marsula, Lamont, & Wissel, 1998; Talluto & Benkman, 2013, 2014).

Prior research shows that red squirrels (Fig. 1) select against serotiny by differentially preying on seeds in serotinous lodgepole pine cones, thereby depleting the arboreal seed bank (Talluto & Benkman, 2014). Moreover, cone serotiny is polygenic and heritable (Fig. 1B, C; Parchman et al., 2012) and thus should respond to selection. When red squirrel densities exceed one and half per hectare, past modeling suggests that selection by squirrels against serotiny overwhelms the selective pressure from fire favoring serotiny in Yellowstone (Talluto & Benkman, 2014). At lower squirrel densities, the balance between selection by fire and squirrels sets the frequency of serotiny (Talluto & Benkman, 2013, 2014); variation in the relative intensities of selection by fire and squirrels accounts for the spatial variation in serotiny across Yellowstone. Results from this study also lend insight into why the occurrence of serotiny is uniformly around 90% in isolated ranges where squirrels are not present east and west of the northern Rocky Mountains (Talluto & Benkman, 2014)—a frequency of serotiny that exceeds that found in areas with squirrels (Benkman & Siepielski, 2004). In fact, following a fire in the Cypress Hills, east of the Rocky Mountains, where red squirrels did not occur and 92% of the trees are serotinous (Benkman & Siepielski, 2004), the postfire density of lodgepole pine seedlings was 2,500,000/ha (Newsome & Dix, 1968)—vastly greater than postfire sites in Yellowstone where red squirrels are present (Turner et al., 2004).

**Figure 3:**
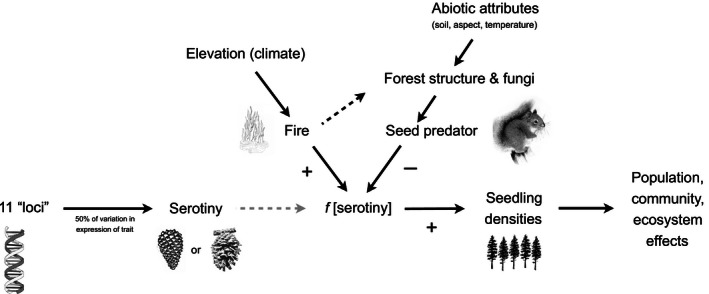
A schematic of the genes to ecosystems, including the two main opposing selective agents on the frequency of serotiny: fire, which favors serotiny, and seed predation by red squirrels, which selects against serotiny. The density of red squirrels is related to attributes such as the amount of clay in the soil and forest structure, where the latter is related to abiotic features such as soil bulk density, mean annual temperature, and stand age. Because red squirrel densities increase with stand age, reductions in fire intervals act to reduce red squirrel densities (black dashed arrow) and further shift the balance of selection resulting in higher frequencies of serotiny. Conversely, increases in fire intervals result in higher average squirrel densities, further shifting the balance of selection resulting in lower frequencies of serotiny. Figure modified from Talluto and Benkman (2014)

The models in Talluto and Benkman (2014) assumed that local densities of red squirrels were stable over successive fire intervals and pine generations, an assumption supported by the match between predicted and observed levels of serotiny. We ignore annual variation in squirrel density, because red squirrels are strongly territorial resulting in relatively stable local densities especially within Rocky Mountain lodgepole pine forests (Gurnell, 1984; Smith, 1968). Our goal here was to determine whether environmental (e.g., climate, topography, soil; e.g., Hahm, Riebe, Lukens, & Araki, 2014) and stand structure attributes are related to spatial variation in densities of red squirrels. An earlier analysis included climate, topography, and forest structure, but did not include soil characteristics (Talluto & Benkman, 2013). Because soil affects forest growth and structure (Schoenholtz, Van Miegroet, & Burger, 2000), and the occurrence of soil fungi (Paul & Clark, 1996)—an additional and important food source for red squirrels (Fletcher et al., 2010; Smith, 1968)—inclusion of soil characteristics should enhance our ability to predict squirrel densities based on environmental factors that are stable across pine generations. Such information will help bridge established linkages between the genes underlying serotiny (Parchman et al., 2012) and the ecosystem consequences of spatial variation in the frequency of serotiny (Turner, Romme, & Tinker, 2003; Turner et al., 2004, 2016). Such results would be directly applicable across ~26 million ha of Rocky Mountain lodgepole pine forests in the United States and Canada (Lotan & Critchfield, 1990) and would provide insight into the link between ecological and evolutionary processes from genes and phenotypic traits to biotic and abiotic interactions to landscape structure and function (Fig. 3).

## 2 Methods

We focused our research on 12 low‐elevation sites established by Talluto and Benkman (2013, 2014), because here fire frequency is high (historical fire intervals of 135–185 years compared to 280–310 years at high elevations; Schoennagel et al., 2003), yet variation in serotiny is substantial and is inversely correlated with the population densities of red squirrels (Talluto & Benkman, 2013). In our analyses, we used the environmental variables in Talluto and Benkman (2013) that had relatively high predictive value for red squirrel density: habitat variables: canopy cover, and mean and coefficient of variation (CV) of diameter at breast height (DBH) of all trees, and also included stand age; topographic variables: extent of orientation toward north (northness) and toward the east (eastness) of the sites. We also calculated in ArcGIS 10.1 several climatic variables (30‐year mean precipitation, and maximum, mean, and minimum temperature) using PRISM (PRISM Climate Group 2012), and several topographic variables (aspect, surface curvature, heat load index, and compound topographic index, which is a measure of moisture availability and a function of slope and upslope contributing area) using Jeff Evan's Geomorphometric Toolbox (Evans, Oakleaf, Cushman, & Theobald, 2014). In August 2014, we collected known volumes of mineral soil at 0–20 cm below the litter and organic layers using an impact‐driven core sampler at 20 randomly selected locations at each of the 12 sites. The samples were air‐dried for 4–8 days, then sealed in plastic bags, and brought back to the University of Wyoming where the proportion of each sample made up of clay, sand, and silt was estimated using an hydrometer, and bulk density g/cm^3^ was measured. We used the same core sampler to determine the depth (0–50 cm) of the restrictive horizon as a measure of soil depth at each location. We then used the mean values of the 20 samples from each site to characterize each site; median for soil depth was used because many values exceeded 50 cm and were recorded as 50 cm. Table 1 provides mean values for many of the variables for the 12 study sites.

**Table 1:** Summary statistics for the 12 study sites

Site name	Red squirrel density (no./ha)	Proportion *Pinus contorta*	Mean DBH (cm)	DBH CV	Stand age (years)	Proportion clay	Bulk density (g/cm^3^)	Mean temp (°C)	Surface curvature
Arnica	1.25	0.82	23.7	34.7	265	0.03	0.728	0.82	−0.246
Bechler	0.42	0.76	22.9	33.8	156	0.17	0.472	2.25	0.031
Delacey	1.04	0.90	25.4	40.8	273	0.04	0.694	0.54	−0.092
Hayden	1.61	0.96	23.5	43.3	367	0.05	0.677	0.75	0.092
Madison 0	0.31	1.00	17.0	31.3	130	0.05	0.639	2.14	0.000
Madison 1	0.71	1.00	19.6	30.8	122	0.03	0.597	2.15	0.000
Nez Perce 2	0.47	1.00	17.1	34.4	140	0.13	0.751	1.36	−0.061
Nez Perce 3	0.54	1.00	19.2	38.4	302	0.15	0.678	1.32	−0.031
Nez Perce 4	0.62	1.00	17.4	31.5	140	0.03	0.789	1.54	0.061
Nez Perce 44	0.42	1.00	17.2	36.2	292	0.03	0.668	1.34	−0.031
Polecat	1.25	0.98	18.6	37.8	209	0.07	0.600	2.13	0.061
Weasel	1.04	0.98	22.8	44.2	295	0.11	0.689	0.54	−0.092

We used generalized linear models (GLM) to determine which set of environmental variables best predicts squirrel density as measured by Talluto and Benkman (2013) at each of the 12 sites, assuming a Gaussian error distribution and using a log‐link function (to correct for 0‐truncation and nonhomogeneity of errors in squirrel density). We used a nonparametric bootstrap to estimate confidence limits for regression parameters. For each model, we resampled the data with replacement and recomputed the regression parameters; this procedure was repeated 1,000 times with the 95% bootstrap confidence limits taken as the 2.5% and 97.5% quantiles of the resulting distribution. We used Akaike information criterion (AICc for small sample sizes) to compare all possible models with three or fewer variables (no interactions included) and present the models with ΔAICc < 4 from the best model (i.e., the model with the lowest AICc value). Some explanatory variables were highly correlated (specifically, maximum and mean temperature, and DBH and soil depth; Fig. S1). To avoid problems from collinearity, we excluded from consideration models including two variables with a correlation coefficient >0.75 or variance inflation factors exceeding 2. Our goal was to assess whether variables, whose variation among sites is likely to be stable across pine generations, account for variation in squirrel density. One of the independent variables in the best model accounting for squirrel density is likely to be stable among tree generations (percent clay in soil), whereas the temporal stability of variation in the other independent variable (CV of DBH) is less certain. Consequently, we used GLM as above to compare all possible models with three or fewer variables to further examine whether abiotic features, that were likely temporally stable across pine generations, can explain variation in CV of DBH. Path analysis would have been an ideal method; however, our sample size of 12 sites prevented its use. Consequently, our results should be interpreted as hypotheses requiring further testing.

## 3 Results

The best model included the CV of DBH and the proportion clay as predictors of squirrel density (ΔAICc = 0; *R* ^2^ = .64; *p *=* *.01; Table 2A). The only other model that included two or more variables and had a ΔAICc < 4 was the model that included the CV of DBH, proportion clay, and eastness (ΔAICc = 2.81; *R* ^2^ = .78, *p *=* *.014; Table S1). In both these models, squirrel density increased with increasing variation in DBH and decreasing clay content of the soil; the latter model indicated that squirrel density increased when the aspect of the site was oriented toward the east (Table S1). Several models with only a single independent variable had ΔAICc < 4 (Table S1), including the CV of DBH (ΔAICc = 0.54, *R* ^2^ = .43, *p *=* *.023), mean DBH (ΔAICc = 1.44, *R* ^2^ = .42, *p *=* *.035), soil depth (ΔAICc = 3.02, *R* ^2^ = .39, *p *=* *.074), mean and maximum temperatures (ΔAICc = 2.39, *R* ^2^ = .37, *p *=* *.055 and ΔAICc = 2.83, *R* ^2^ = .38, *p *=* *.068, respectively), and stand age (ΔAICc = 1.46, *R* ^2^ = .37, *p *=* *.039). Squirrel densities increased with the mean and variation in DBH, increases in soil depth and stand age, and decreases in mean and maximum temperatures (Table S1).

**Table 2:** The parameter estimates for the best models (A) accounting for variation in red squirrel density among the 12 low‐elevation sites in Yellowstone National Park (*R* ^2^ = .64 [95% CI: 0.26, 0.93], *p *=* *.01), and (B) accounting for variation in the CV of the diameter at breast height (*R* ^2^ = .72 [95% CI: 0.46, 0.91], *p *=* *.0004). (C) The best model accounting for variation in the CV of the diameter at breast height using environmental variables unlikely to vary over time (*R* ^2^ = .87 [95% CI: 0.59, 0.99], *p *=* *.002)

Parameter	Estimate (95% CI)
A. Best model accounting for variation in red squirrel density	
Intercept	−2.74 (−5.06, −1.43)
Percent clay	−4.92 (−10.44, −0.98)
CV of DBH	0.077 (0.043, 0.134)
B. Best model accounting for variation in CV of DBH	
Intercept	26.14 (23.56, 28.87)
Stand age	0.046 (0.030, 0.062)
C. Best model using less temporally variant variables accounting for variation in CV of DBH	
Intercept	68.39 (47.58, 91.59)
Bulk density	−28.69 (−58.24, 0.07)
Mean temperature	−8.79 (−12.98. −5.93)
Surface curvature	19.84 (−1.56, 99.25)

The model having the smallest AICc with CV of DBH as the dependent variable (ΔAICc = 0) included only stand age (*R* ^2^ = .72, *p *=* *.0004; Table 2B). Older stands had greater variation in DBH. This would cause within‐location temporal variation in red squirrel density and can account for why squirrel density increases with stand age (above), but would not lead to consistent spatial variation in squirrel density. Focusing on environmental variables that would be consistent in a location from one fire interval to the next, and potentially cause consistent variation among stands, the best model predicting CV of DBH included soil bulk density, mean temperature, and surface curvature (*R* ^2^ = .87, *p *=* *.002; Table 2C). Models with CV of DBH as the dependent variable and ΔAIC < 4 relative to the previous model (Table S2) included mean temperature and bulk density (ΔAICc = 0.21; *R* ^2^ = .74, *p *=* *.003), mean temperature and surface curvature (ΔAICc = 2.24; *R* ^2^ = .69, *p *=* *.007), and mean and maximum temperature (ΔAICc = 1.39, *R* ^2^ = .55, *p *=* *.006 and ΔAICc = 2.96, *R* ^2^ = .49, *p *=* *.013, respectively). The CV of DBH increased with decreases in both soil bulk density and mean temperature, and increases in surface curvature and stand age (Table 2B, C).

## 4 Discussion

Our models, including just two or three environmental variables, explained a substantial proportion of the variation in red squirrel density among lodgepole‐dominated forests in Yellowstone and provided a substantial improvement in explanatory power over the model in Talluto and Benkman (2013; 64% vs. 33% of the variation). Moreover, these models, with the exception of CV of DBH, included factors that are unlikely to change over time at a given location (e.g., soil texture, such as percent clay; Schoenholtz et al., 2000). The other variable in the models accounting for red squirrel density, namely variation in tree size at a site (CV of DBH), increases with stand age, presumably because of gaps formed from tree mortality and subsequent infilling; most trees in young stands will have germinated soon after a fire, so DBH should be relatively homogeneous initially. A model that incorporated abiotic factors (soil bulk density, mean temperature, and surface curvature) unlikely to vary with stand age, and are likely to contribute to consistent spatial differences among locations, also accounted for a large proportion of the variation in DBH (Table 2C). Thus, red squirrel density will vary at a site as the stand ages after a fire, and among sites because of spatial variation in at least several abiotic factors. Importantly, we can account for spatial variation in red squirrel density based on features of the environment that would establish stable spatial patterns of selection exerted by red squirrels against serotiny. This in turn accounts for a considerable amount of the spatial variation in serotiny across the Yellowstone Plateau (Talluto & Benkman, 2013, 2014), with dramatic population, community, and ecosystem consequences (Turner, Romme, Reed, et al., 2003; Figs 2 and 3).

The increase in red squirrel density with stand age should reinforce selection on serotiny that is associated with variation in fire regime. Shorter fire intervals favor an increase in the occurrence of serotiny because individual trees are more likely to experience a stand‐replacing fire and benefit from an accruing arboreal seed bank, and shorter fire intervals should result in lower densities of red squirrels when a fire occurs and thus reduce the intensities of selection exerted by squirrels against serotiny. Conversely, longer fire intervals reduce the advantages of serotiny (an individual tree is more likely to die from other causes than fire and thus not benefit from producing serotinous cones) and result in higher densities of red squirrels when fire eventually occurs, and therefore, selection exerted by squirrels against serotiny will be more intense. Thus, a decrease in fire intervals has a negative indirect effect on average densities of red squirrels (Fig. 3).

Red squirrel densities declined with increasing amounts of clay in the soil, presumably because clay being colloidal strongly binds water, reducing its availability (i.e., reducing the soil's water potential) for both plants and fungi (Brady & Weil, 1999; Paul & Clark, 1996). Clay is also associated with small pore sizes that prevent plant root and fungal growth (Paul & Clark, 1996). Consequently, increasing amounts of clay could lead to poorer plant and fungal growth (Paul & Clark, 1996), lower fungal abundance and diversity (Talley et al., 2002), and reduced ability of fungi to produce sporocarps (Krebs, Carrier, Boutin, Boonstra, & Hofer, 2008), which red squirrels feed heavily on in addition to conifer seeds (Fletcher et al., 2010; Smith, 1968). Pines might further benefit from increased mycorrhizal abundance and diversity (Paul & Clark, 1996) and as a consequence have more resources to allocate to cone production.

Red squirrels were more abundant with increasing variation in the size (DBH) of the trees. Red squirrel density also increased with increases in the mean size of the trees. A diverse forest structure might permit easier movements between the ground and trees or among trees, or it could be related to pine seed production. For example, the number of lodgepole pine cones produced increases exponentially as tree diameter increases (Smith, 1968). Consequently, the total number of cones and seeds produced per unit area, which is an important measure of territory quality for red squirrels (Kemp & Keith, 1970; Smith, 1968), should increase with both increases in mean tree size and with an increasing CV in tree size. A positive second derivative for the increase in cones with tree size (Smith, 1968) ensures that the overall level of cone production will increase with increasing tree size variation for a given mean tree size (i.e., Jensen's inequality; Ruel & Ayres, 1999). Forest structure could also affect vulnerability to predation; however, we do not know whether, for example, DBH or variation in DBH affects predation on red squirrels. In contrast, correlative studies and field experiments show food supply limits the abundance of red squirrels (Kemp & Keith, 1970; Klenner & Krebs, 1991; Smith, 1968; Sullivan, 1990).

The CV in DBH increased with decreases in soil bulk density and mean temperature, and increases in surface curvature. Soil bulk density is an important characteristic of soil. Soil porosity increases with decreases in bulk density and represents improved growing conditions by creating a better environment for root and fungal hyphae growth, and increased aeration and water filtration (Brady & Weil, 1999; Harris, Young, Gilligan, Otten, & Ritz, 2003; Paul & Clark, 1996). Thus, increasing variation in DBH could reflect better growing conditions and greater plant and fungal (sporocarp) productivity, and presumably better habitat for red squirrels. The importance of feedbacks between soil, fungi, and lodgepole pine (Binkley & Giardina, 1998; Paul & Clark, 1996) and whether such feedbacks alter the densities of red squirrels are unknown. Regardless, such interactions are unlikely to alter the stability of spatial variation in red squirrel densities and are unlikely to cause red squirrels to be common in one location during one fire‐free interval and relatively scarce during the next interval. Increasing positive values for surface curvature apparently result in increasing variation in DBH. Positive values of surface curvature reflect convex surfaces (e.g., hills and ridges), while negative values reflect more concave surfaces (e.g., depressions and valleys). Why greater variation in DBH occurs on ridges than in valleys, as well as why variation in DBH increases with decreases in mean temperature, is uncertain.

In summary, several environmental features that vary among lodgepole pine forests in Yellowstone account for a substantial amount of the variation in the density of red squirrels. With the exception of stand age, which is an important variable influencing stand structure (CV of DBH) within a location over time, these features of the environment, including the percent clay and bulk density of the soil, and landscape orientation, are unlikely to vary locally from one fire‐free interval to the next, and thus the spatial variation in red squirrel densities at a given phase in stand development is likely to be relatively stable over time. This will lead to consistent local and spatially variable patterns of selection exerted by red squirrels against serotiny. Furthermore, this helps justify the assumption of locally stable red squirrel densities in the model by Talluto and Benkman (2014) and helps account for the match between their model's predictions of the frequency of serotiny and observed. Although the actual environmental features determining red squirrel densities require further study and are likely to vary away from the lava flows of the Yellowstone Plateau, we suspect that in Yellowstone and elsewhere, red squirrel densities have been locally stable in forests of a given age from one fire interval to the next. If so, then, a small squirrel will have a spatially variable evolutionary impact with outsized ecological consequences across a vast area of the Rocky Mountains.

## Funding Information

Our research was funded by the Robert Berry Chair Endowment.

## Conflict of Interest

None declared.
