# Supplementary figures and images for: From the ground up: biotic and abiotic features that set the course from genes to ecosystems
Source: Ecol Evol. 2016 Sep 9;6(19):7032–8. doi: 10.1002/ece3.2468 (PMC5513219; doi:10.1002/ece3.2468)

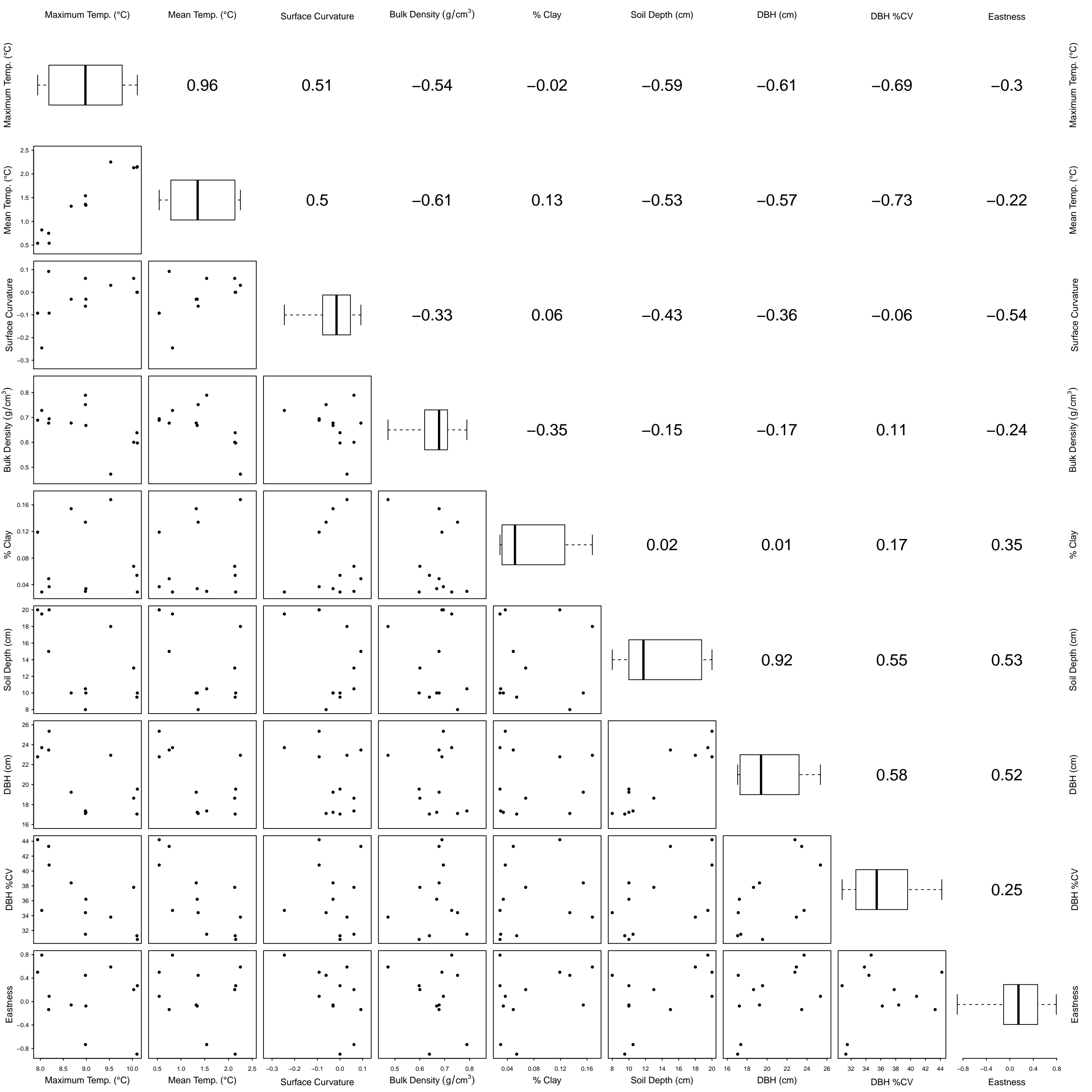

Supplement: Supplementary file 1 [file ECE3-6-7032-s001.pdf]
