# Supplementary material for: From the ground up: biotic and abiotic features that set the course from genes to ecosystems
Source: Ecol Evol. 2016 Sep 9;6(19):7032–8. doi: 10.1002/ece3.2468 (PMC5513219; doi:10.1002/ece3.2468)
Supplement: Supplementary file 2 [file ECE3-6-7032-s002.docx]

Appendix

Appendix Fig. 1. Pairwise relationships between explanatory variables. Boxplots on the diagonal show the median and quartiles of each variable (boxes) along with the data range (whiskers). Values in the upper triangle are correlation coefficients between each pair of variables. Only variables that were included in models with AICc < 4 are shown.

Appendix Table 1. The coefficients (95% confidence limits in parentheses below) of the predictor variables for the top models (∆AICc < 4) accounting for variation in the densities of red squirrels at 12 study sites in Yellowstone National Park.

____________________________________________________________________________________________________________

Maximum Mean Percent Soil depth DBH CV of DBH Eastness ∆AICc

temperature temperature clay

____________________________________________________________________________________________________________

-4.92 0.077 0

(-10.44, -0.98) (0.043, 0.134)

0.072 0.54

(0.026, 0.12)

0.10 1.44

(0.03, 0.18)

-0.48 2.39

(-1.07, -0.01)

-6.15 0.075 0.40 2.81

(-9.88, 0.383) (0.027, 0.158) (-0.46, 1.19)

-0.41 2.83

(-0.95, 0.06)

0.054 3.02

(0.017, 0.091)

____________________________________________________________________________________________________________

Appendix Table 2. The coefficients (95% confidence limits in parentheses below) of the predictor variables for the top models (∆AICc < 4) accounting for variation in the coefficient of variation in DBH at 12 study sites in Yellowstone National Park.

______________________________________________________________________________

Maximum Mean Surface Bulk ∆AICc

Temperature temperature curvature density

______________________________________________________________________________

-8.79 19.84 -28.69 0

(-12.98, -5.93) (-1.56, 99.25) (-58.24, 0.07)

-7.44 -29.42 0.21

(-10.46, -3.58) (-82.75, -10.79)

-5.16 1.39

(-7.82, -1.74)

-6.62 20.57 2.24

(-11.17, -3.04) (-24.89, 58.40)

-3.86 2.96

(-6.65, -0.76)

______________________________________________________________________________
